# Proteogenomic convergence for understanding cancer pathways and networks

**DOI:** 10.1186/1559-0275-11-22

**Published:** 2014-06-01

**Authors:** Emily S Boja, Henry Rodriguez

**Affiliations:** 1 Office of Cancer Clinical Proteomics Research, National Cancer Institute, National Institutes of Health, 31 Center Drive, MSC 2580, 20892 Bethesda, MD, USA

**Keywords:** Protein identification and quantitation, Post-translational modification, Targeted proteomics, Proteogenomics, Protein networks, Signaling pathways, Mathematical and computational modeling, Omics integration, Systems biology

## Abstract

During the past several decades, the understanding of cancer at the molecular level has been primarily focused on mechanisms on how signaling molecules transform homeostatically balanced cells into malignant ones within an individual pathway. However, it is becoming more apparent that pathways are dynamic and crosstalk at different control points of the signaling cascades, making the traditional linear signaling models inadequate to interpret complex biological systems. Recent technological advances in high throughput, deep sequencing for the human genomes and proteomic technologies to comprehensively characterize the human proteomes in conjunction with multiplexed targeted proteomic assays to measure panels of proteins involved in biologically relevant pathways have made significant progress in understanding cancer at the molecular level. It is undeniable that proteomic profiling of differentially expressed proteins under many perturbation conditions, or between normal and “diseased” states is important to capture a first glance at the overall proteomic landscape, which has been a main focus of proteomics research during the past 15-20 years. However, the research community is gradually shifting its heavy focus from that initial discovery step to protein target verification using multiplexed quantitative proteomic assays, capable of measuring changes in proteins and their interacting partners, isoforms, and post-translational modifications (PTMs) in response to stimuli in the context of signaling pathways and protein networks. With a critical link to genotypes (i.e., high throughput genomics and transcriptomics data), new and complementary information can be gleaned from multi-dimensional omics data to (1) assess the effect of genomic and transcriptomic aberrations on such complex molecular machinery in the context of cell signaling architectures associated with pathological diseases such as cancer (i.e., from genotype to proteotype to phenotype); and (2) target pathway- and network-driven changes and map the fluctuations of these functional units (proteins) responsible for cellular activities in response to perturbation in a spatiotemporal fashion to better understand cancer biology as a whole system.

## Introduction

Recent advances in high throughput genomics in the past few years as evidenced by large-scale collaborative initiatives such as The Cancer Genome Atlas (TCGA), the International Cancer Genome Consortium (ICGC), Therapeutically Applicable Research to Generate Effective Treatments (TARGET) and others have extensively characterized and sequenced the genomic alterations from different types of cancer [1-6]. These highly coordinated efforts are creating large complex datasets for mining and computational modeling by the scientific community. The availability of these rich datasets to the public sets the stage for understanding the underlying mechanisms of cancer initiation and progression, and the development of new, more effective targeted cancer interventions. It is well known that aberrations in genome structure and defects in maintenance and repair are instrumental for tumor initiation and progression by expediting the accumulation of favorable genotypes in evolving premalignant cells [7-9]. As such, genome aberrations and instability are clearly enabling characteristics that are causally associated with the acquisition of many cancer hallmarks [10]. In addition, cancer is a highly heterogeneous disease in terms of cell type and tissue origin, and a disease of dysregulation of multiple pathways involved in fundamental cellular processes, such as death, proliferation and differentiation [11-13]. Hence, a better understanding of cancer biology using deep genomic and proteomic characterization of high quality human clinical specimens, well-annotated cell lines and animal models (+/- perturbation) is the first logical step to pursue, perhaps even at the single cell level [14].

Following the maturity of genomic sequencing technologies, proteomic technologies such as mass spectrometry (MS), protein- and affinity-based arrays and bead-based flow cytometry have also progressed and evolved tremendously over the past 15 years to deeply and systematically characterize the human proteomes [15-18]. The combination of brute force protein profiling and targeted proteomic methodologies for a subset of the proteomes or selected pathways have proven to be powerful approaches for identifying and quantifying proteins in biological materials, such as tumors, proximal fluids and blood. As these advances in multi-omics technologies yield large inventories of genes, transcripts, proteins, metabolites and relevant biological information with data and bioinformatic tools becoming publicly accessible, the paradigm has gradually shifted from studying single genes and proteins in a specific biological system to the study of cellular processes as a whole (systems biology). A fundamental insight in signal transduction, as recently precipitated by omics results, has demonstrated that cancer-related pathways are not linear but are rather organized as networks [19]. As an extremely complex and heterogeneous disease, cancer inevitably displays highly nonlinear dynamics due to the involvement of a large number and variety of components that interact via complex networks. The challenge for the research community in this multi-omics era is to solve the puzzle of how these network modules work synergistically to regulate the processes whereby cells respond to external and internal signals. This will in turn shed light on these complex biological phenomena by generating detailed roadmaps of a variety of cellular networks based on many types of data. Once the network models are established by bioinformatic and statistical tools, our hope is to translate gained knowledge from research to medicine and clinical practice, for example, knocking out several target molecules in several biochemical pathways for a more effective cancer therapy due to the fact that cells often find alternative molecular routes to escape the blockage targeted by a single drug.

Herein, we provide a comprehensive proteogenomic-centric review on the current status of technologies and bioinformatics for studying cancer systems biology in light of the growing maturity of high throughput, deep genomic sequencing and deep proteomic characterization. Additionally, we offer our perspective on the importance of developing and implementing an efficient, community-based multi-omics integration pipeline with standards to further this area of science. Altogether, the emerging multi-omics era promises a collection of high throughput, multidimensional quality data and computational resources from which new knowledge and hypotheses can be generated and validated to enhance the understanding of signaling events and roadmaps critical for cancer development and progression.

### Overview on biochemical and proteomics technologies for the analysis of pathways and networks

#### Studying known pathways and networks using quantitative proteomics and PTM-omics

With tremendous improvements in instrumentation and automation, particularly with MS during the last 15 years, shotgun proteomics have been widely used to detect a large number of proteins (often in the range of 1000 s to 10,000 s depending on the biological matrices) and their PTMs such as phosphorylation. Mass spectrometry (MS)-based labeling methodologies, such as iTRAQ or stable isotope labeling by amino acids in cell culture [SILAC]) and/or label-free approaches have made semi-quantitative protein measurements, including PTMs and isoforms feasible at a global scale [20,21]. Although these approaches do not reveal direct protein-protein interactions (PPIs) for mapping interactomes, the interpretation of proteomics and PTM-omics data generated from these studies can be performed through established known protein-protein interaction pathways and networks, or through the inference of regulatory networks built upon a variety of “omics” input. The inference of regulatory networks is usually done by transcriptomics studies initially, and would benefit from the addition of rich proteomics data in order to enhance pathway predictions with the goal of understanding the regulatory pathways rather than directly mapping them. One advantage of using proteomics data for this is unarguably the added value from quantitative measurements of PTMs and functional sub-proteomes (e.g., kinase/phosphatase families) important for signaling events throughout the networks that neither genomics nor transcriptomics can provide. Furthermore, signaling pathways modulate cellular processes interact such that the resulting signal crosstalk contributes largely to many of the key characteristics of cancer or other dysfunctional cells [22-24]. In some cases, PTMs crosstalk and work in concert to determine the final biological read-outs (e.g., crosstalk between histone phosphorylation and acetylation [25]). Thus, quantitative assessment of various types of PTMs involved in the regulation of cellular processes becomes crucial to the understanding of signal flow in relevant pathways and networks.

In this regard, Beli et al. reported a multi-level proteomics study that integrates large-scale quantitation of protein PTMs and protein abundances in response to DNA damage signals [26]. In the past, proteomic studies have been biased towards phosphorylation analysis of ataxia telangiectasia, mutated (ATM), ATM- and Rad3-related (ATR), DNA-dependent protein kinase (ATM/ATR/DNA-PK) substrates or nuclear proteins, attributing to the fact that DNA strand breaks were primarily triggered by the phosphatidylinositol 3-kinase-like protein kinase (PIKK) ATM and, by the related kinases ATR and DNA-PK to a lesser extent. These PIKKs preferentially phosphorylate substrates at serine or threonine residues, followed by a glutamine ([S/T]Q motif), setting them apart from other protein kinases in terms of substrate specificity. However, complex DNA damage response (DDR) signaling is not confined to the nucleus, and involves a plethora of PTM mechanisms including phosphorylation, acetylation, ubiquitination and sumoylation along with transcriptional and post-transcriptional regulation [26]. Quantitative measurements of DDR-regulated phosphoproteome, acetylome, and proteome have broaden our knowledge of DNA damage signaling networks and highlighted an important link between RNA metabolism and DNA repair.

Despite the intricacy of pathway crosstalk modulated by different PTMs, phosphoproteomics remain one of the most important and preferred PTMs to study by biologists as it is believed that quantitative determination of phosphorylation sites and their stoichiometries are possible indicators of kinase activity and substrate specificity. One such approach is to comprehensively localize and quantify phosphopeptides in a complex biological mixture with and without perturbation to infer upstream kinase activities using MS [27]. Complementarily, kinase assays linked with phosphoproteomics (KALIP) have been developed for the determination of downstream substrate specificity and the identification of direct substrates of protein kinases with high sensitivity. Kinase assays linked with phosphoproteomics (KALIP) is based on a kinase reaction using formerly in vivo phosphorylated peptides as candidates [28]. This method efficiently improves the sensitivity of a kinase reaction linked to endogenous phosphoproteomics modulated by the kinase of interest to detect substrates. With this strategy, it has been demonstrated that spleen tyrosine kinase (Syk), a 72-kDa protein tyrosine kinase (RTK) with duel properties of an oncogene and a tumor suppressor in distinctive cell types (known to play a crucial role in adaptive immune receptor signaling, particularly in B cells), used as a target kinase, helped identify direct substrates of Syk specific to B cells and breast cancer cells. As a result, both known and unique substrates, including multiple centrosomal substrates for Syk were identified, supporting a unique mechanism that Syk negatively affects cell division through its centrosomal kinase activity [28]. Similar activity-based methodologies have been developed using functional protein microarray platforms to identify kinase substrates representing a broad spectrum of different biochemical functions and cellular roles [29]. These array-based methodologies have recently been applied to profile PTMs, including phosphorylation, ubiquitination, acetylation, glycosylation and nitrosylation.

Although many methodologies using proxies for kinase activities (e.g., antibody- or MS-based measurements of phosphorylation states upon biological perturbation) [30,31] are very useful and quantitative, these indirect inferences of kinase activity generally lack a temporal component, making the interpretation of enzymatic reaction rates very difficult. Additionally, other types of PTMs occurring on kinases as previously described play a role in modulating their activities, which are usually not accounted for in these studies. To complicate this issue further, there is a lack of correlation between phosphorylation events and kinase activities, thus arguing that it may be better to directly measure kinase activity rather than a substitute. Consequently, Activity-based Protein Profiling (ABPP) (or chemoproteomics) has been developed to address this issue. The use of chemical probes to directly measure enzyme activity is not a new concept. They usually consist of two key elements (a reactive group for binding and covalently labeling the active sites of a given enzyme class or classes; and a reporter tag for the detection, enrichment and identification of probe-labeled enzymes in proteomes), such as fluorescence or MS [32,33]. These ABPP probes selectively label the active forms of enzymes followed by quantitative analysis to characterize changes in enzyme activity without corresponding changes in protein expression. However, recent improvements in such technologies, such as click chemistry, have been applied to more extensively study sub-proteomes of a complex biological context rather than focusing on one single enzyme. An example of “click chemistry” version of the ABPP method is the analysis of the functional state of enzymes in living cells and organisms [34], an improvement to in vitro assays. Contrary to traditional ABPP, this version allows the profiling of living cells and organisms by treating these specimens with tag-free azide- or alkyne-modified probes, followed by conjugation in vitro to the complementary alkyne- or azide-modified tags via cycloaddition reaction to visualize probe-labeled proteins. This approach has enabled the capture and characterization of several enzyme classes, including many that have central roles in cancer such as kinases and phosphatases [35,36], histone deacetylases [37], and deubiquitylases [38], followed by subsequent multiple reaction monitoring mass spectrometry (MRM-MS) quantitation. Hence, this version of activity-based probes enhances the elucidation of underlying mechanisms of disease pathophysiology and potential therapeutic intervention as analysis can reveal the intricate interplay between different signaling transduction pathways responsible for cellular function such as differentiation and apoptosis. Similarly, biotin-tagged acyl-phosphates of ATP and ADP have been developed, which are capable of acylating the conserved active site lysines for a range of known human protein and lipid kinases and other ATP-dependent enzymes, followed by LC-MS analysis post affinity enrichment of biotinylated peptides on streptavidin beads. This broader, probe-based strategy was deployed to profile well-studied kinase inhibitors against >200 kinases in native cell proteomes, revealing biological targets for some of these inhibitors with therapeutic potential. Such activity-based studies truly highlight the complexities of protein kinase behavior in the cellular context [39].

Complementary to MS detection, Stains et al. developed a probe in which a phosphorylation-sensitive fluorescent amino acid, Sox, a sulfonamido-oxine fluorophore, has been employed to directly monitor kinase activity in unfractionated cell lysates [40]. With this approach, phosphorylation at a proximal residue can dramatically increase the affinity of Sox for Mg^2+^, resulting in fluorescence increase, a method applicable for substrates for all kinases. A second-generation cysteine derivative of Sox fluorophore (CSox) has been engineered, allowing for the incorporation of N- and C-terminal kinase recognition elements to improve selectivity and kinetic properties with lower sample demand. Using a panel of probes including p38α, mitogen-activated protein kinase-activated protein kinase 2, extracellular-signal regulated kinases 1/2 (ERK1/2), Akt and protein kinase A, this method has been used to perform activity measurements of individual kinases in a model of skeletal muscle differentiation and cancer tissue samples, providing direct, quantitative readouts of kinase enzymatic activity associated with cellular differentiation and human tumors [41]. More importantly, this proof-of-principle study can be expanded to larger sample sizes to illustrate biological perturbations in kinase activities in a given disease.

#### Mapping direct protein-protein interactions

To complement data from shotgun proteomics and PTM-omics studies discussed in the previous section, PPIs can be directly mapped by more focused sample isolation strategies—particularly the use of tandem affinity purification (TAP)–tagged proteins. This method is based on the creation of a fusion protein with a tag, expressed in cells and used as a bait to purify stable protein complexes that assemble on the TAP-tagged protein *in vivo*, followed by SILAC or other labeling strategies (e.g., iTRAQ)-MS to identify proteins that interact with particular signaling networks. This sensitive and specific method has demonstrated its utility in large-scale protein interaction mapping in lower organisms (e.g., *Drosophila melanogaster*[42] and yeast [43]), and elucidating smaller interactomes and signaling pathways in mammals [44]. Finally, the TAP–MS approach applied to transgenic mice enables the comparison of protein complex organization between different tissues, facilitating the characterization of novel interacting partners not previously identified in cell cultures [45]. This method can be used for real determination of protein partners quantitatively *in vivo* without prior knowledge of complex composition, is simple to execute and often provides high yield. However, the tags may obscure binding of a new protein to its interacting partners, affect protein expression levels, and not be sufficiently exposed to the affinity beads, thus skewing the results.

In addition to TAP-MS, the yeast two-hybrid (Y2H) system has long been applied to enhance the mapping of direct PPI networks with vast improvement and optimization over the years. For instance, Y2H maps of human mitogen-activated protein kinase (MAPK) signaling network not only confirmed many known interactions but also revealed many new roles for chaperons and proton pumps in the regulation of MAPK functions [46]. Furthermore, Y2H interaction data, in combination with time-resolved proteomic data on protein phosphorylation induced by epidermal growth factor (EGF), tracked the dynamic information flow in the EGF-activated ERK network, a member of the MAPK family [47]. This allowed the identification of several hitherto 18 unknown modulators of EGF-stimulated ERK signaling.

Despite vast improvements in such methodologies over the years, the caveats of both of these experimental approaches still remain, including: (1) using model organisms readily manipulated genetically (e.g., expression of a bait protein, RNA interference screening) with the assumption that interactions observed in these model systems reflect normal physiology and are meaningful to human biology; (2) false-positive hits yielded by the Y2H system suffer from the absence of known PPIs that depend on contextual information (e.g., PTMs that may or may not occur in yeast); (3) a lack of dynamic changes in PPIs do not reveal the flow of signaling information. Furthermore, unlike Y2H, TAP-MS may fail to detect transient interactions, low stoichiometric protein complexes, and/or those interactions occurring only in certain physiological conditions under-represented in exponentially growing cells as most cellular processes require PPIs, or the assemblies of large protein complexes that are dynamic and assemble in spatial and temporal manner to store and relay various cellular signals or to contribute to the cellular architecture (e.g., enzymes often interact with regulatory subunits required for their activity, or subcellular localization [48,49]). Although monitoring changes in protein interactions in response to signals or over a time course of stimulation can track the flow of a signal through a network [50-52], the high cost and time limit for the generation of dense time-course data required for reconstructing large-scale temporal signaling dynamic networks can greatly burden the researchers. As an alternative approach to relieve such burden, one can design smaller-scale experiments to interrogate a subset of known pathways in a time-resolved manner, or one or more PTMs and key network hubs. With this compromise, proteomic measurements of time-dependent changes in signaling pathways can be obtained using targeted, multiplexed and quantitative approaches, such as MRM-MS coupled with stable isotope dilution (SID) [53-55], *in vitro* kinase assays [56], quantitative immunoblotting and enzyme-linked immunosorbent assays (ELISAs) [57] or protein arrays [58-60]. By monitoring dynamic changes in these PPIs, temporal data have been used to reconstruct signaling pathways involved in cell differentiation and apoptosis [61-63]. In addition, immuno-enrichment of phosphotyrosine residues and quantitative MS methods have previously explored time-dependent changes in signaling downstream of epidermal growth factor receptor (EGFR) [64,65]; the combination of MS, phosphorylation motif–directed antibodies, and phosphorylated serine-threonine–binding modules (e.g., 14-3-3 proteins or the Polo-box domain of Polo-like kinases) identified signaling networks involved in cell migration, metabolism, mitosis, and DNA damage [66-68]; and the use of analog-specific protein kinase mutants and MS identified comprehensive lists of substrates, e.g., those previously unknown to cyclin-dependent kinase 1-cyclin B, with the potential of expanding our understanding of kinase-substrate connections in signaling networks [69,70].

In reality, different technologies/platforms for studying and refining known pathways in databases and literature based on proteomic signatures, or for directly mapping protein interaction networks have their own advantages and disadvantages, thereby arguing for the benefit of complementary approaches to better answer the biological questions under investigation. While some of these advantages and disadvantages have previously been described, it is also important to add that direct mapping of PPIs are mostly done in cell or tissue culture systems that can easily be genetically manipulated, or perturbed by biochemical agents in a time-dependent fashion to measure the temporal dynamics of signaling circuitries. In contrast, a human tissue sample, e.g., tumor biopsy materials during a time-course drug treatment clinical trial (perturbation), while representing true human cancer biology, are more difficult to acquire and control due to clinical practice guidelines, pre-analytical variables, etc. These samples are more suitable for pathway and network analyses through deep proteogenomic characterization, followed by bioinformatic analysis using known PPI pathways and networks, or inferring regulatory networks from a variety of “omics” data. In that regard, a lack of analytical sensitivity of direct MRM-MS to quantitatively measure targeted signaling proteins (especially phosphorylated proteins) in tiny amounts of tumors, which often populate most personalized therapeutic trials, is also a major limiting factor. It often requires affinity reagents or other depletion/separation approaches to enrich for the proteins/peptides of interest prior to MS analysis. On the other hand, while multiplexed reverse-phase protein arrays (RPPAs) or ELISAs provide more sensitive detection of proteins involved in signaling events even with small amount of materials (e.g., from laser-capture microdissection to enrich for cancer cells), it requires high quality antibodies and some level of prior knowledge on protein interactions under investigation that may not exist in the marketplace and database/literature, respectively.

### Overview on bioinformatics for the analysis of pathways and networks

#### Multi-dimensional cancer omics studies and related data

Over the past 10–15 years, the genomics community has produced large amounts of high quality datasets and revolutionized the understanding of biology and medicine. High throughput technologies, such as next-generation genome sequencing, RNAseq, chip-on-chip, large-scale chromatin immunoprecipitation (ChIP-seq) microarrays, have been applied to measure gene expression levels and gene regulatory elements that identify genes with influence on some interesting phenotypes on a genome-wide scale. These technological advances in deep sequencing (Ion Torrent’s PGM, Illumina MiSeq, and Applied BioSystem’s SOLiD to name a few) have facilitated a paradigm shift in biological studies from a ‘one gene model’ to a ‘multiple gene model’ and have generated many large-scale biology projects (e.g., human genome project [71]). As these technologies become more affordable and accessible, the implementation of such large-scale projects will become more routine in both research and perhaps clinical settings. For instance, comparative genomics and microarray-based approaches have generated methods such as the mathematical simulation of pathway dynamics that enabled the reconstruction of gene regulatory networks to understand and predict how different transcription factors interact to activate or deactivate defined sets of genes. Using a database of known and predicted transcription factor binding sites (TFBS), all genes with a specific motif pair combination in their promoters have been identified. Subsequently, the expression coherence score calculated based on microarray data for each set of genes associated with a TFBS pair yields a quantitative measure for the synergistic behavior of TF combinations [72].

On the cancer front, the success of large-scale genomics projects have anchored on a set of statistically-powered number of human specimens, including thousands per cancer type characterized by TCGA from the National Cancer Institute (NCI) and National Human Genome Research Institute (NHGRI), ICGC, and TARGET. These projects are significantly advancing the comprehensive characterization of cancer genomes for understanding cancer at DNA (genomics) and RNA (transcriptomics) level, and facilitating the discovery of molecular targets and translation of those findings into the clinic. Such deep analyses included multiple platforms, e.g., whole tumor genome sequencing, miRNA expression, RNAseq, mutations, and DNA copy number that provide rich datasets for the scientific community to mine and extract meaningful biological information (hypothesis generation) [73,74]. As a result, genomic alterations associated with cancer have been produced through multi-dimensional datasets that include high level integrative analysis with omics datasets. As an example, TCGA research network has comprehensively cataloged the molecular aberrations in 487 high-grade serous ovarian cancers. While the initial report from TCGA on 489 high-grade serous ovarian adenocarcinomas (487 of which had corresponding miRNA data) presented a broad molecular picture of the disease, microRNAs, gene copy number alterations and methylation of gene promoter regions that globally influence gene expression ultimately determine cellular behavior [75]. In-depth analyses revealed four ovarian cancer transcriptional subtypes, three microRNA subtypes, four promoter methylation subtypes and a transcriptional signature associated with survival duration, which illuminated on the impact of BRCA1/2 (BRCA1 or BRCA2) and CCNE1 aberrations on patient survival. Pathway analyses of ovarian cancer data suggested the defect of homologous recombination in half of the analyzed tumors, and implicated the role of NOTCH and FOXM1 signaling in serous ovarian cancer pathophysiology. Analysis of miRNAs within the TCGA ovarian dataset (particularly miRNA and mRNA data) generated from the same set of tumors demonstrated miRNAs as a key factor with huge impact on gene expression in ovarian cancer. Due to these public datasets, other research groups have now been able to develop computational algorithms using TCGA multi-analysis data and two additional datasets based on methods that identify network alterations and quantify network behavior through gene expression [76]. As a result, a network biomarker candidate around the platelet-derived growth factor (PDGF) pathway that significantly stratifies survival rates in ovarian cancer patients, which the expression levels of single or sets of genes alone cannot explain the prognostic stratification, emerged, further strengthening the power of gene expression networks. Collectively, the TCGA Pan-Cancer project assembled data from thousands of patients with primary untreated tumors occurring in different sites of the human body, covering 12 tumor types including GBM, head and neck squamous carcinoma (HNSC), lung adenocarcinoma (LUAD), breast carcinoma (BRCA), ovarian carcinoma (OV), colon adenocarcinoma (COAD), etc. Six types of omics characterization were performed (including RPPA data), tying the data elements across the various platforms by the fact that the same samples were used for each to maximize the potential of integrative analysis. Data integration enables the identification of general trends, including common pathways, revealing master regulatory hubs activated or deactivated across different tissue types [77].

**Figure 1:**
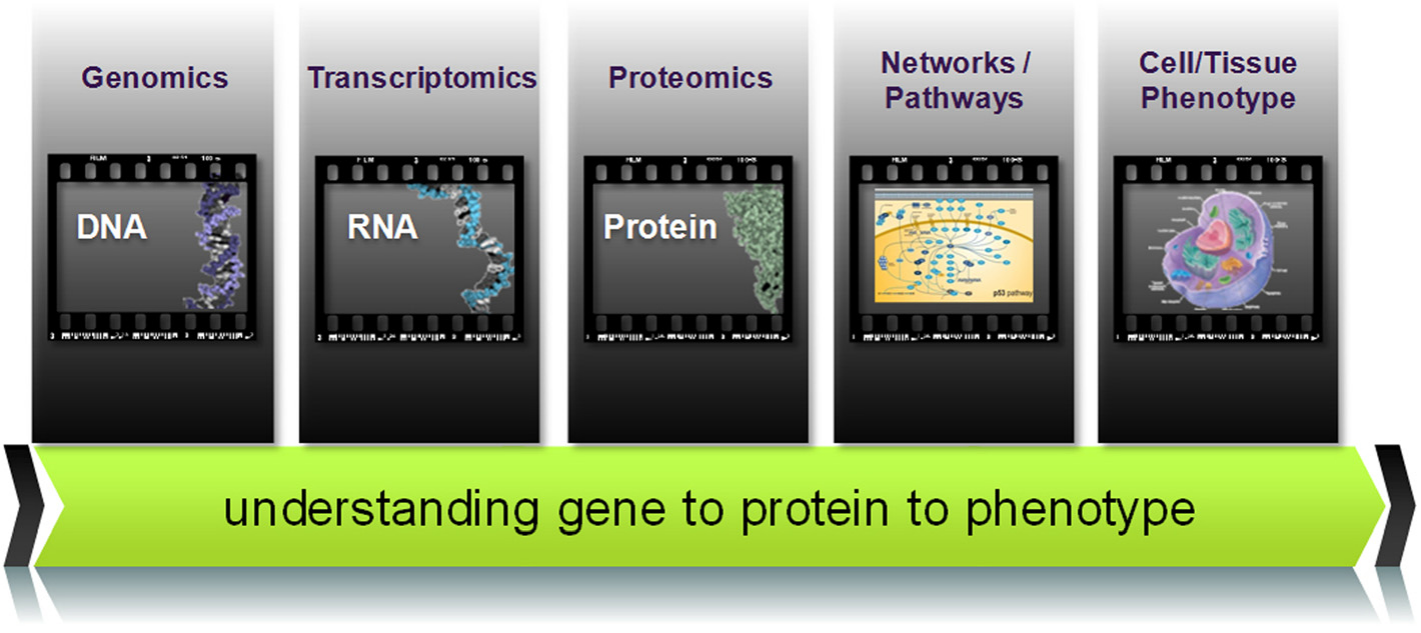
**Linking cancer genotypes to cancer phenotypes.** The comprehensive molecular level analysis at the DNA, RNA, protein and dynamic protein pathways and networks through proteogenomics and network modeling can greatly enhance our understanding of cancer systems biology (i.e., linking genotype to proteotype to cell/tissue phenotype).

What does this entail for proteomics and integrative biology? These rich datasets present an unprecedented opportunity for the research community to study cancer systems biology by linking cancer genotype to cancer phenotype through the understanding of cancer proteotype and the complex dysregulated signaling pathways and interaction networks. An example of the importance of such integrative analysis is shown by Wang, *et al*. where the corroboration of genomic aberrations at the protein level has been demonstrated with KRAS in pancreatic cancer in which targeted MRM-MS approach coupled with immunoprecipitation of intact RAS protein isoforms detected a single point mutation at the peptide level in KRAS oncogenes from a cell line, tumor sample and pancreatic cyst fluid at sensitivity of <25 fmol/mL [78]. While peptide-level mutation confirmation is crucial, the impact of such activating mutations on pancreatic cancer proteome was not evaluated. This requires a comprehensive characterization of wild-type and mutant KRAS proteomes including PTMs and subsequent integrative analysis of omics data superimposed on genes/proteins and their regulatory networks, signaling pathways and dynamics of operation. As illustrated in Figure 1, a systematic and comprehensive molecular-level characterization (DNA, RNA and protein) could provide a specific context for important biological processes responsible for cell and tissue function, thereby shedding new light on how genomic instabilities and aberrations result in changes in dynamic protein signaling pathways and networks to give rise to its ultimate phenotypic behaviors.

**Figure 2:**
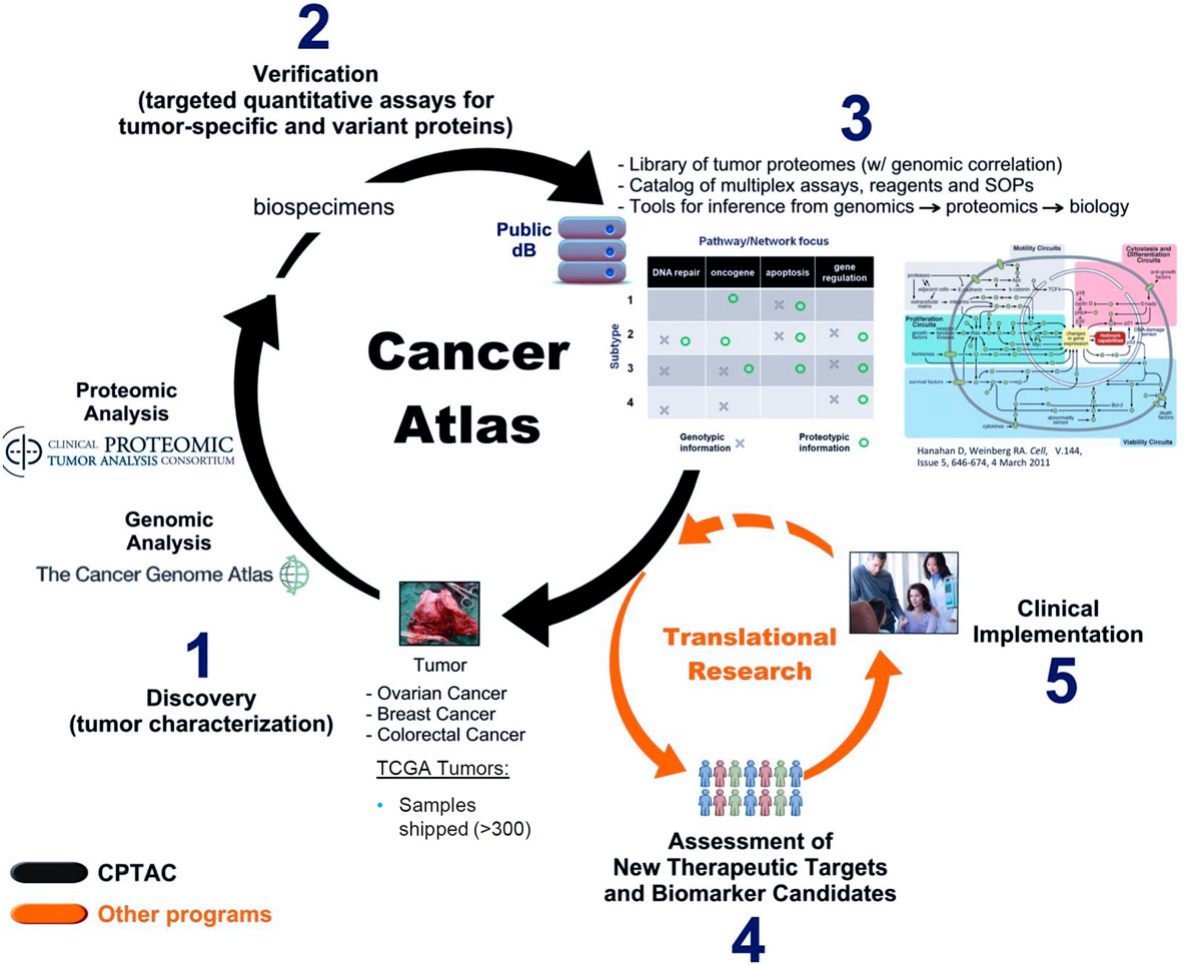
From proteogenomics to cancer biology – An integrative pipeline for building a comprehensive human cancer atlas from discovery to validation.

To begin connecting these levels of biology, a multi-disciplinary, network-driven program, Clinical Proteomics Tumor Analysis Consortium (CPTAC) (http://proteomics.cancer.gov), a component of the Clinical Proteomic Technologies for Cancer initiative (CPTC) at the NCI, was launched in 2011 to capitalize on the investments made by large-scale cancer genomics initiatives. It is believed that changes deriving from genetic alterations can be functionalized by comprehensive proteomic analysis on the same tumor to enhance the rich multi-dimensional genomic data sets, improve the understanding of cancer biology, and potentially drive the development of new diagnostics and therapeutics. The scientific approaches to this endeavor are illustrated in Figure 2, where a set of statistically powered, genomically-characterized tumors, for example, by TCGA and other sources are proteomically characterized in a Discovery stage using MS and array technologies (1), followed by integrative proteogenomics to add new insights to cancer biology at the network and pathway level, providing prioritized proteins of interest for targeted proteomic assay development and testing in a separate cohort in a Verification stage (2). Simultaneously, genomic information can direct targeted proteomic measurements including mutations and isoforms without prior proteomic characterization as a complementary method. The goals for this proteogenomic initiative in the next few years as illustrated in (3) are the production of community resources including: 1) data and databases of tumor-specific proteomes with proteogenomic annotation/genomic correlation (https://cptac-data-portal.georgetown.edu/cptacPublic/); 2) improved understanding of cancer systems biology (for further hypothesis generation and validation), along with associated tools and methods; and 3) verified protein targets with a catalog of multiplexed, quantitative assays to measure them, including standard operating procedures (SOPs), associated data and necessary reagents (http://assays.cancer.gov; and http://antibodies.cancer.gov). CPTAC is complementary to existing efforts, such as the Centers for Cancer Systems Biology (CCSB) of The Integrative Cancer Biology Program (ICBP) at the NCI (http://icbp.nci.nih.gov/), and the human proteome organization (HUPO)’s vision to unite the Encyclopedia of DNA Elements (ENCODE) Consortium (http://encodeproject.org) [79] (funded by the NHGRI) with the chromosome-centric human proteome project [80], both of which decipher the ‘parts list’ of the human body [80]. By realizing the tremendous value that can be gained from integrative omics, the scientific community is committed to making progress towards the implementation of efficient strategies for developing, applying and standardizing technologies and tools to improve our understanding of diseases.

#### Integrative proteogenomics: benefits and challenges

The importance for omics data integration in understanding biology has been demonstrated by studies with multidimensional datasets at the DNA, RNA and protein levels. One such example stems from the important role of EGFR signaling in epithelial cell regulation and cancer biology where data representing RNA regulation, protein abundance and protein phosphorylation were combined to investigate and better describe the mitogenic response of human mammary epithelial cells to EGF using multiple datasets from whole genome microarrays, MS-based proteomics and large-scale western blots using more than 1000 antibodies for protein phosphorylation [81]. Systematic analysis of the practical benefits of merging heterogeneous time-dependent data for networks and pathways concluded the major processes and signaling networks known to be regulated by EGFR in this cell type, while individual data sets from different types of platforms provided different views of EGFR-induced cell processes and pathways qualitatively. As demonstrated in this study, one of the important reasons for integrative analysis is that RNA abundance changes are not always a good predictor of protein abundance changes, especially over a time scale of several hours. Although the canonical correlation analysis described in this study (with a correlation coefficient r of 0.44 between RNA and protein expression for 199 genes) is generally in agreement with correlations previously reported at single time points [81], less than half of the protein abundance changes measured by high resolution MS were accompanied by corresponding RNA changes even when time course data are included, strongly suggesting the involvement of a high degree of post-transcriptional regulation in the response of mammalian cells to EGF. Some clusters of RNA and protein pairs in this study showed a classical pattern of post-transcriptional regulation, where RNA changes preceded or coincided with a corresponding change in protein abundance, while other clusters indicated complex patterns that imply feedback processes between protein half-life and compensatory RNA induction. Thus, estimating steady-state mRNA and protein changes from a single time point can be misleading as a result of the time needed for protein synthesis and degradation, while temporal-based analyses of correlations between global protein and gene expression patterns in human cells could be more accurate. Additionally, each analytical platform was biased towards observed cellular processes, supporting that the networks derived from heterogeneous datasets via data integration can show a more connected topology than those derived from a single dataset. These studies have conclusively shown the power of mapping the dynamics of PTM networks in tremendously improving our understanding of signal propagation through the pathways, and providing added value to biology from which one single omics dataset cannot derive.

Using cancer genomics data, it is likely to map the functional modules and the cancer-driving mutations onto network modules, each of which can be a subnetwork itself containing the functionally-linked pathways that reflect many cancer hallmarks [10]. For instance, an integrative analysis using network reconstruction and co-expression module identification-based approaches on the human signaling network and cancer driver-mutating genes has revealed network modules of cell cycle and apoptotic pathways that are critical for all types of cancer [82]. WNT/TGF-beta cross-talk, WNT/VEGF signaling and MAPK/focal adhesion kinase pathways have been identified as targets of rare driver mutations in breast, colorectal cancer, and glioblastoma, respectively. To go beyond cancer biology, a one-person (Dr. Michael Snyder) longitudinal omics study was published using a combination of genomics, transcriptomics, proteomics (including autoantibody profiles) and metabolomics approaches on collected blood samples. Although this proof-of-principal study has demonstrated the promise of detailed omics profiling in providing molecular and physiological information of medical significance with the potential to change the future for personalized health monitoring and medicine [83], multi-omics studies on larger cohorts are needed to demonstrate added value of omics data to current medical practice for a specific intended clinical use.

**Figure 3:**
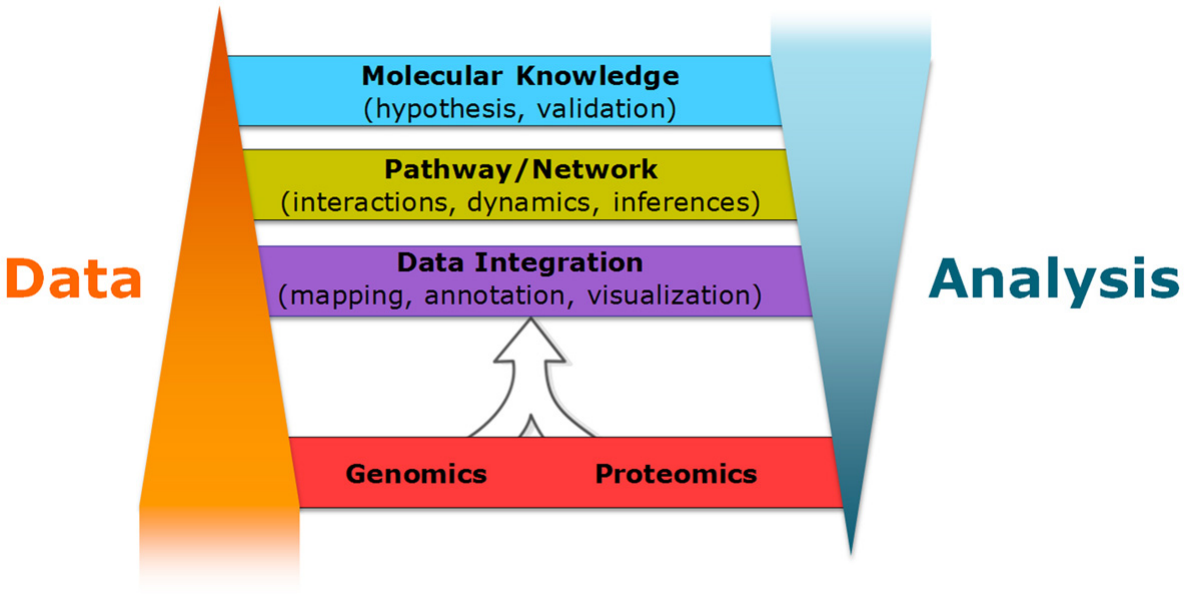
**Climbing up a proteogenomic data ladder.** Integrative omics experiments generate tiers of data and knowledge to improve cancer systems biology. Proteogenomic data accumulate at the lower tiers of the data ladder (proteogenomic mapping of linear sequences and protein expression and PTM changes due to genomic alterations), and compress as data analyses become more labor-intensive, complex, and multi-dimensional at the network and pathway level (upper tiers).

As high throughput approaches generate mountains of data, the global research community is coalescing around the overwhelming realization to improve data management and interpretation in order to obtain better insights into molecular mechanisms and biological principles from these data. The fundamental understanding of biology is, in large part, based upon the understanding of genes and their encoded protein products. Hence, the mapping of cancer genomes and proteomes arising from the cancer genomes can provide valuable information on the effects of genomic aberrations on the functional units of a cell. In the case of TCGA-CPTAC proteogenomics data and other similar datasets, the primary information gleaned from the convergence of both types of data is the proteogenomic mapping against a human reference genome (e.g., HG19) to better define genome annotation [84,85], to confirm and discover peptide-level detection of genomic aberrations such as single mutations [78] and splice variants, and to assess the effect of genomic aberrations on global protein expression and PTM alteration [86] (Figure 3, lower tiers). Currently, identifying unannotated genes and verifying gene calls, defining translational start/stop sites as well as reading frames, and describing signal peptide processing events and PTMs, are feasible tasks that can be carried out at a full-genomic scale once the genomic sequence becomes available [87,88]. This approach has demonstrated its efficiency in discovering existing misannotations and enriching genome annotation in organisms, such as *Yersinia*[89] and *Candida glabrata*[90]. Furthermore, proteomic data has been utilized to examine the translation of RNAs to proteins on a genome-wide scale using computational tools to map peptide-based MS data to their encoding genomic loci (genome-based peptide fingerprint scanning) [91] and PTMs by protein inference engine [92]. Through the melding of several large and heterogeneous data sources including MS-based proteomics data, genome sequences, gene annotation sets, and single nucleotide polymorphism sets, this approach revealed alternatively spliced or frameshifted translation products that could not be easily discovered by standard proteomics database search strategies.Establishing the most comprehensive protein list is an essential prerequisite prior to analysis of the quantitative cellular dynamics of proteins, their PTMs and complex network interactions and interpretation of these data as a whole, which constitutes the basis for systems biology. Through network inference, analysis and modeling, it will ultimately provide biological insight by identifying dysregulated networks and pathways as a result of genomic alterations in cancer (Figure 3, upper tier). This will further facilitate new hypothesis generation and experimental validation studies. As more data accumulates through genomic, transcriptomic and proteomic characterization, data integration and analysis become more labor-intensive and complex requiring more sophisticated computational tools while climbing up the data ladder.

#### Network analysis and modeling

Realistically, the only efficient way to handle large amounts of omics data and the relationships within those datasets buried underneath its surface is through mathematical representation and computation. The integration of many types of omics data and the development of effective computational tools to decipher complex systems is believed to be one of the best approaches to leverage the tsunami of data for biological insight in a comprehensive way. The integration of various types of networks composed of different types of signaling components, however, remains an extremely daunting task, partially due to the fact that protein networks are typically interaction or modification networks with a time component (temporal distribution). While transcriptional networks resemble protein networks in this respect, the time delay between the production of mRNAs and their encoded protein products can vary significantly between different genes. To complicate this matter, bottom up proteomics commonly used to identify and quantify proteins based on their proteolytic peptide fragments make it difficult to match proteins to their splice variants easily detectable by transcriptomic experiments. The mathematical methods for analyzing these networks are also fundamentally different. Altogether, these issues make diverse datasets difficult to be directly superimposed onto networks. One possible solution is to compare the interpreted information content from different technologies instead of primary data, but rather the structure of the inferred networks and the functional predictions resulting from them. Semantic Web tools are one of such methods that can automatically connect different types of information already available in various databases and repositories by logical rules. However, it requires rigorous validation of the computational models and their underlying data [93,94].

Currently, the understanding of how complex molecular and cellular outcomes that control the fate of a cell arise from the dynamic interaction topologies at the mechanistic level remains poor. Constructing a series of networks with temporal data, therefore, can reveal the dynamics of biological processes on a time scale such as tumor progression, with the advantages of simplifying complex interactions and allowing for the identification and quantitation of relationships between signal inputs and outputs. To reach these goals, network construction relies heavily on integrative omics data and accumulated knowledge that begin with combining diverse datasets into networks using informatics tools. Some of these computational tools merge information from different databases and allow text-based searches from the literature into a single Web resource including STRING [95] and iRefWeb [96], while others attempt to define network topology as the weighted collection of the available evidence for specific PPIs or both. For instance, a number of online databases and tools allow the construction and analysis of weighted collections of protein networks including BioGRID [97], BIND [98], MINT [99] and DIP [100]. Kyoto Encyclopedia of Genes and Genomes (KEGG) [101], Pathway Commons [102] and Reactome [103] are publicly accessible resources for pathway models. Once network construction pieces together functional networks that reflect the relationships between genes and proteins under certain conditions, e.g., cancer gene signaling networks in metastasis, these networks can elucidate the links between omics data and the fundamental processes of cancer development and metastasis on a time scale. As a critical part of network analysis, visualization tools allow the researcher to “see” molecular interaction networks and integrate these interactions with omics data. Some network visualization examples include Cytoscape (http://www.cytoscape.org) at the cBio Cancer Genomics Portal, an open source bioinformatics software with visualization capability and additional features available for network and molecular profiling analyses, new layouts, additional file format support and connection with other databases [104], and VisANT (http://visant.bu.edu/) [105], another network visualization tool designed for the integrative data mining of multi-scale network/pathways that features Network Module Enrichment Analysis (NMEA) and GO Term Enrichment Analysis (GOTEA) in batch mode. Most recently, NetGestalt has been developed (http://www.netgestalt.org) to provide a data integration framework based on the context of a biological network by allowing simultaneous presentation of large-scale experimental and annotation data from many different platforms (e.g., DNA microarray, MS-based proteomics, RNA-seq) [106]. The unique feature of NetGestalt is its ability to integrate any type of data in linear tracks, allowing researchers to visualize and hypothesize based on protein pathways and networks. The Cancer Proteome Atlas (TCPA) links TCGA genomics data with RPPA proteomics data, also providing visualization of networks based on TCGA cancer types (http://app1.bioinformatics.mdanderson.org/tcpa/_design/basic/index.html).

Following network construction and visualization, computational analysis of the constructed networks using mathematical and statistical modeling tools takes place. Once the overall network connectivity has been established, the next logical step is to understand how signals functionally propagate through a network, largely due to the dynamics of PTMs, including phosphorylation, ubiquitination and acetylation. This remains a huge technical challenge in itself due to the complication caused by misidentification of certain post-translationally modified peptides, and thus missing certain network components. To model network dynamics with time course data, the inference of the network architecture based on a quantitative definition of the connectivity between various nodes (or modules) becomes extremely important. A variety of mathematical and computational models have been established for the analysis of network inference that range from data-driven modeling [107] using multiple clustering analysis (e.g., K-means, hierarchical, or self-organizing maps). For example, time course studies of phosphoproteomics data have been clustered to reveal signals with temporal dynamics [108,109] and regression analysis such as principal component analysis-based [107], Bayesian inference methods [110], Boolean networks [111], Modular Response Analysis (MRA) [112], and differential equation modeling [113,114].

High throughput data, albeit rich and powerful, tend to suffer from low information content (e.g., the observable results contain little information about the unknown parameters causing them). An example is when a local perturbation initially confined to a particular network can propagate and cause widespread global changes in the network, thereby masking immediate connections and routes. This issue is particularly pertinent to large omics datasets as their snapshots of the cellular states arise from a variety of interactions throughout cellular networks, even in response to a single local perturbation. Hence, the choice of applying the correct modeling approaches depends, in a large part, on how much mechanistic detail is needed in the model by researchers provided with the available experimental data. Since all modeling tools have their own limitations, it might be beneficial to deploy a combinatorial approach in order to compensate for the limitations of individual methods. Alternatively, MRA, a method that takes advantage of the modular nature of biological systems to infer network architecture and the strengths of connections between nodes from network responses to systematic perturbations can serve to deduce information without prior knowledge on reaction stoichiometries and kinetics [115]. In this case, a module can either be a single network including a gene, its mRNA and encoded protein product, or distinct sets of proteins or network components with defined functionalities. The effect of a change in a module on the activity of another module via these connectivities with other modules kept constant during perturbation needs to be quantitatively characterized. This combinatorial approach has demonstrated its utility in inferring dynamic topology of feedback loops in the MAPK cascade in cells activated with EGF via the organization of differently phosphorylated protein species and isozymes of the MAPK cascade into modules [116].

A recent study where a large fraction of mutations in pancreatic cancer were shown to be associated with a core set of signaling pathways base on microarray techniques could be a good candidate for proteogenomics-based network analysis [117]. This study showed that genes in those signaling pathways have a tendency to be overexpressed, suggesting their potential as clinically useful biomarker candidates [117]. However, this study did not establish whether overexpression of pathway-specific genes resulted in a detectable increase in the levels or activities of the corresponding proteins. This presents an opportunity for integrative proteogenomics to (1) identify changes in pathway-specific protein abundance or PTMs; (2) further verify such pathway-specific proteogenomic targets by targeted proteomics, e.g., MRM-MS or protein arrays prior to network modeling. Further analysis can go beyond the pathway-association approaches by building cell-specific network topologies to link cancer-specific mutations to mechanistically-linked protein biomarker candidates. Such integrative approaches could potentially provide a mechanistic framework to predict pathways and proteins associated with a particular cellular state [118] that would likely benefit from more omics datasets. In fact, others have demonstrated the utility of integrative computational modeling in a number of studies based on quantitative experimental data on molecular and cellular networks to enhance our understanding and prediction of cancer. Some of these published studies pertain to the analysis of gene expression profiles to identify markers correlated with metastasis, resulting in more reproducible subnetwork markers than individual marker genes selected without network information and achieving higher accuracy in the classification of metastatic versus non-metastatic tumors [119], the identification of dysregulated pathways with respect to tumor phenotype in comparison to normal, the elucidation of oncogenic mutation consequences that affect cell behavior by changing cellular networks, and the identification of novel targets in regulatory networks for more effective cancer therapies [120]. Specifically in a study on ErbB receptors using human mammary epithelial cells with increased expression level of ErbB2, systems biology approaches identified nine phosphorylation events from MS data that serve as important “network controls”, including phosphatidylinositol-4,5-bisphosphate 3-kinase (PI3K) that enabled the prediction of cell behavior [121] (as reflected in Figure 1: linking genotype to phenotype). Moreover, combined therapeutic strategy against both EGFR and c-MET targets enhanced the effectiveness as demonstrated by the phosphoproteomic analysis of cells expressing increasing levels of the constitutively active EGFRvIII mutant, suggesting cross-activation of c-MET pathway by EGFRvIII, a mutant frequently identified in tumors [122].

Although the cancer systems biology strategies are not entirely new, their not-fully-realized potential will help build realistic network models of tumors (via network construction), as well as identifying network modules and the key genes and other network features in each module from these networks (via network analysis and modeling). To ensure its success, it is imperative for researchers to realize that (1) while network construction from databases such as Pathway Commons provide aggregated knowledge of biological networks under diverse conditions (i.e., neither disease-specific nor sample-specific), the mounds of omics data generated using complementary proteogenomic technologies to date are often sample-specific, revealing the unique characteristics of that particular sample (and the disease), such as the TCGA-CPTAC datasets. Therefore, in order to reach meaningful biological conclusions representative of the disease population, it inevitably requires statistically-powered number of samples that may or may not be available to researchers. In this regard, a combinatorial approach to use both prior knowledge from pathway and network databases and experimental data in the context of the specific biological/disease conditions might be useful to help refine known information and shed new light on the biology; (2) the results derived from the system biology approaches must ultimately be validated experimentally (e.g., using siRNA) in cancer cell lines and/or mouse models (e.g., progression models for different cancer stages) or statistically (e.g., Bayesian approaches that accommodate missing data points and calculate the degree of uncertainty), or by refining the inferred network using a combination of omics data [123]. An example of this stepwise process that integrates experiment and computational data has been demonstrated in the elucidation of a complex regulatory network that governs the activity of the mammalian target of rapamycin complex 2 (mTORC2) signaling network [124]. It is not trivial to validate these models, however, especially when systematic perturbation of network nodes is involved while inferring large networks (e.g., from global transcriptomics or proteomics data due to a large number of experiments required). Despite these challenges, at least for the validation of networks inferred from proteomics or Y2H data, targeted proteomic data from MRM-MS [125] or protein arrays [57,58] and subsequent integration of proteomic data with large-scale RNAi-mediated protein knockdowns can make the systematic exploration and validation of large parts of the inferred networks feasible. For instance, the ERK interactome maps from large-scale Y2H screens including 18 previously unknown modulators of EGF/ERK signaling were experimentally validated by RNAi knockdown of components in cells [126], generating networks with high confidence that revealed unknown pathways and signaling modulators that influenced ERK activity.

#### Building a proteogenomics computational pipeline and experimental standards

Lastly, challenges and roadblocks to the success of integrative omics biology for the generation of new and improved molecular knowledge remain in various stages of a multi-omics pipeline. Specifically, such issues include but are not limited to a lack of systematic assessment of the impact of pre-analytical variables especially with clinical samples (e.g., ischemic time during tumor procurement, a variety of sample processing and storage conditions, etc.) on the integrity of proteins and their modifications [127,128]; a lack of experimental study design to reduce bias and strengthen statistical rigor of sample size [129,130], and the complication from tumor and population heterogeneities and their effects on omics analysis in cancer [131,132]; and a lack of better technologies and standardization of protocols with high quality reagents [133]. Computationally, there is a lack of efficient data storage, more automated network visualization/modeling tools (with built-in statistical analysis and standardization) to streamline the nature of intrinsic complexity of multi-omics data analysis across all possible datasets, and to make it simple enough for biologists to interpret the data. This argues for a harmonized and consolidated resource (e.g., a consolidated database, standardized gene names) that can provide added value to the global scientific community, similar to the world-wide Protein Data Bank for three-dimensional structures [134].

Such an endeavor requires a long-term commitment of community-based approaches. Recognizing the benefits of information and resource sharing in building an enterprise of high quality, multidimensional data, along with their assays, reagents and protocols for public access, the scientific community has already made significant progress in the last several years in two aspects with respect to data generation and data analysis. These include the generation of well-characterized monoclonal antibodies (http://antibodies.cancer.gov; http://neuromab.ucdavis.edu; http://proteincapture.org/) [135]; MS-based peptide spectral libraries, databases and datasets (http://peptide.nist.gov/; http://www.peptideatlas.org/; http://www.srmatlas.org/; https://cptac-data-portal.georgetown.edu/cptacPublic/, etc.) [136-138], informatics tools for targeted MRM-MS assay design [139,140] and qualification criteria (http://assays.cancer.gov), as well as data analyses and computational modeling [106,141-143]. While it is impossible to list all databases and tools, some publicly available resources for data integration, pathway visualization and analysis are PathGuide [141] (http://www.pathguide.org), BIOPAX (http://www.biopax.org) [142], cBio (http://cbio.mskcc.org/) [143], MAPT and PAICE (http://sourceforge.net/projects/mapt/ and http://sourceforge.net/projects/paice/) [144], iCTNet as a plugin for Cytoscape (http://www.cs.queensu.ca/ictnet) [145], the Georgetown Database of Cancer (G-DOC) (https://gdoc.georgetown.edu/gdoc/) [146], VANTED (http://vanted.ipk-gatersleben.de/) [147]. Additional tools for the visualization of omics data have been summarized [148]. PathGuide, as an example, provides access to many thousands of pathways and networks that document millions of interactions between proteins, genes and small molecules. BioPAX data standard with well-defined semantics for pathway representation facilitates the integration, exchange, visualization and analysis of biological pathway data by supporting data exchange between data groups. Hence, it allows pathway databases and software to work in unison, enabling the development of pathway visualization from databases and facilitating the analysis of experimentally generated data through the combination of prior knowledge. As omics science moves forward, more efforts should be directed towards building a more efficient omics integration foundation for information storage, flow and exchange to enable the development of quantitative models of biological systems when they can be integrated into a coherent relational network of cellular response.

## Conclusions

High throughput proteogenomic data using deep sequencing technologies emerges as a new era of integrative biology and medicine that could shed new light on the complex, disease-relevant protein interaction networks and signal transduction pathways as a whole system. In combination with time-course perturbation studies, they would greatly enhance the understanding of signal/information flow in response to biological stimuli. With the advent of modern proteogenomic technologies and bioinformatic tools, it becomes increasingly feasible to integrate and visualize such complex types of data by leveraging existing network and pathway knowledge and experimental data, thereby enabling new biological hypothesis generation and experimental validation. This trend will change the research paradigm from more linear and static “one-gene, one protein” models to more complex, functional and dynamic “systems biology” models to complete the biological information flow from DNA to RNA to proteins and to phenotypes. It is important to realize, however, that the success of this endeavor hinges on the success of each component at every stage of the omics data integration pipeline, encompassing correct experimental and statistical study design, understanding the effects of pre-analytical processing of biological and clinical materials, improving analytical reproducibility and accuracy and bioinformatic reliability of network analysis and modeling, visualization and subsequent experimental validation. Only such arduous efforts will ensure that the conclusions can be replicated and modeled after in other laboratories in order to advance our understanding of cancer systems biology.

## Competing interests

The authors declare that they have no competing interests.

## Authors’ contributions

Both authors contributed equally to the writing of this manuscript. Both authors read and approved the final manuscript.
